# Perioperative anaesthetic management of patients with or at risk of acute distress respiratory syndrome undergoing emergency surgery

**DOI:** 10.1186/s12871-019-0804-9

**Published:** 2019-08-14

**Authors:** Denise Battaglini, Chiara Robba, Patricia Rieken Macêdo Rocco, Marcelo Gama De Abreu, Paolo Pelosi, Lorenzo Ball

**Affiliations:** 1 Anaesthesia and Intensive Care, IRCCS for Oncology and Neurosciences, San Martino Policlinico Hospital, Genoa, Italy; 2 Department of Surgical Sciences and Integrated Diagnostics, University of Genoa, Genoa, Italy; 3 Laboratory of Pulmonary Investigation, Carlos Chagas Filho Institute of Biophysics, Federal University of Rio de Janeiro, Rio de Janeiro, Brazil; 4 Department of Anaesthesiology and Intensive Care Medicine, Pulmonary Engineering Group, University Hospital Carl Gustav Carus, Technische Universität Dresden, Dresden, Germany

**Keywords:** Acute distress respiratory syndrome, Perioperative management, Protective ventilation, Emergency surgery

## Abstract

Patients undergoing emergency surgery may present with the acute respiratory distress syndrome (ARDS) or develop this syndrome postoperatively. The incidence of ARDS in the postoperative period is relatively low, but the impact of ARDS on patient outcomes and healthcare costs is relevant Aakre et.al (Mayo Clin Proc 89:181-9, 2014).

The development of ARDS as a postoperative pulmonary complication (PPC) is associated with prolonged hospitalisation, longer duration of mechanical ventilation, increased intensive care unit length of stay and high morbidity and mortality Ball et.al (Curr Opin Crit Care 22:379-85, 2016). In order to mitigate the risk of ARDS after surgery, the anaesthetic management and protective mechanical ventilation strategies play an important role. In particular, a careful integration of general anaesthesia with neuraxial or locoregional techniques might promote faster recovery and reduce opioid consumption. In addition, the use of low tidal volume, minimising plateau pressure and titrating a low-moderate PEEP level based on the patient’s need can improve outcome and reduce intraoperative adverse events. Moreover, perioperative management of ARDS patients includes specific anaesthesia and ventilator settings, hemodynamic monitoring, moderately restrictive fluid administration and pain control.

The aim of this review is to provide an overview and evidence- and opinion-based recommendations concerning the management of patients at risk of and with ARDS who undergo emergency surgical procedures.

## Background

Acute respiratory distress syndrome (ARDS) is a life-threatening condition characterized by hypoxemic respiratory failure and reduced lung compliance [1–3], with parenchymal heterogenicity as demonstrated by CT scan images [4]. ARDS can result from several causes associated with a direct damage to the lung, such as pneumonia, chest trauma with pulmonary contusions, fat embolism, aspiration, and indirect causes, such as: sepsis, pancreatitis, blood transfusions and extra-thoracic trauma [5]. ARDS represents the most severe pulmonary complication after surgery, and is associated mortality rates in the range of 40 to 50% at 30 days from hospitalisation [6]. The incidence of new onset-ARDS in the postoperative period is relatively low, but its impact on patient outcomes is extremely relevant.

### Perioperative management of patients undergoing emergency surgery

**Fig. 1:**
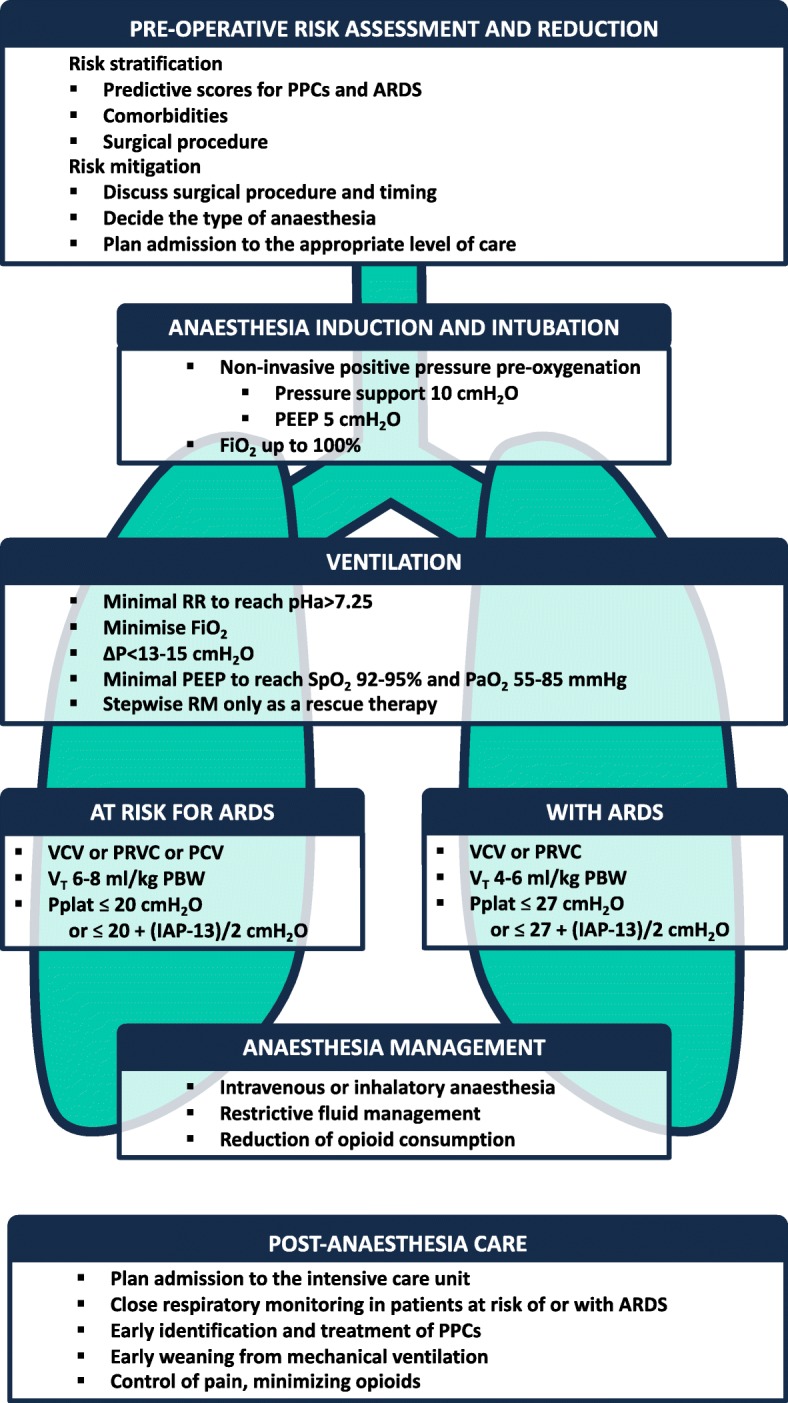
summarises an overview of recommendations concerning the perioperative management of patients at risk of and with ARDS

Patients at risk of and with ARDS must to be promptly identified and managed with specific intraoperative strategies including protective ventilator settings, together with haemodynamic monitorization, the use of specific type of fluids, as well as pain management. Figure 1 summarises an overview of recommendations concerning the perioperative management of these patients**.**

### Preoperative management

#### Patient and surgical-related risk identification

Over the last decade, several scores predicting PPCs and ARDS after surgery have been developed [4–7], but still poorly implemented in the clinical practice [8]. To prevent postoperative ARDS, our ability to identify the surgical population that is at high risk of ARDS is an essential first step. Moreover, delayed recognition of predictive factors for the development of postoperative pulmonary complications is associated with worse outcome [9]. High-risk patients, when correctly identified, require additional monitoring, less invasive surgical procedures, lower risk anaesthesia techniques such as regional anaesthesia when feasible and early planning of intensive care unit (ICU) admission. Preoperative assessment should take into account patient’s basal functional state, comorbidities and the complexity of surgery to minimise the risk of postoperative complications [10, 11]. Centre-specific treatment guidelines may reduce risks and should be discussed with a multidisciplinary team.

The occurrence of PPCs is related to different causes and insults occurring throughout the perioperative period, comprising preoperative patients’ conditions and intraoperative complications [12]. The interaction between predisposing risk factors and surgical and anaesthesiologic management can lead to organ damage translating into postoperative complications. Moreover, during the perioperative period respiratory complications can be further exacerbated by the administration of sedatives, opioids and prolonged supine position. Among others, mechanical ventilation settings and perioperative ventilator management are important parameters linked to the development of PPCs [12–14]. Postoperative respiratory complications increase healthcare costs, because of longer hospital length of stay, unplanned admission to intensive care, hospital readmission and adverse discharge to a nursing home [11].

Scores have been specifically developed to identify patients at risk of ARDS or severe PPCs [83], including the surgical lung injury prediction 2 model (SLIP-2) [15] and the lung injury prediction score (LIPS) [16]. Other scores were generically developed for predicting all-types PPCs [7, 17–19, 83, 84]. It must be underlined that, in the emergency setting, patients who do not have increased risk of PPC when evaluated preoperatively may develop ARDS if desaturation or hemodynamic problems occur during the intraoperative period. Therefore, scores which include intraoperative factors could be more adequate, such as the LAS VEGAS score (Table 1) [18]. The most common complication during surgery is related to the need of hemodynamic support, by the use of vasoactive drugs or increased fluid administration (almost in 30% of patients and more frequently in high risk surgery), followed by respiratory problems, and desaturation [10].

**Table 1:** resumes the principal scores used to predict postoperative respiratory complications

Clinical Variable	Canet J et al. 2010	Gupta H et al. 2011	Arozullah AM et al. 2000	LAS VEGAS investig. 2017	Bauman ZM et al. 2015 [16]	Kor DJ et al. 2011 [15]
Patient dependent						
Age	Yes	Yes	Yes	Yes	No	Yes
Dependent functional status	No	Yes	Yes	No	No	No
ASA score	No	Yes	No	Yes	No	No
Chronic obstructive pulmonary disease	No	Yes	Yes	No	No	Yes
Impaired sensorium	No	No	Yes	No	No	No
Cerebrovascular accident	No	No	Yes	No	No	No
Preoperative SpO_2_	Yes	No	No	Yes	No	No
Transfusion > 4 units prior surgery	No	No	Yes	No	Yes	No
Significant weight loss	No	No	Yes	No	No	No
Preoperative sepsis	No	Yes	No	No	No	No
Preoperative anaemia	Yes	No	No	Yes	Yes	No
Blood urea nitrogen level	No	No	Yes	No	No	No
Recent respiratory infection	Yes	No	No	No	No	No
Relevant alcohol intake	No	No	Yes	No	Yes	Yes
Smoking before operation	No	Yes	Yes	No	Yes	Yes
Chronic steroid use	No	No	Yes	No	No	No
Cancer	No	No	No	Yes	No	No
Obstructive sleep apnoea	No	No	No	Yes	No	No
Hypoalbuminemia	No	No	No	No	No	No
Chemotherapy	No	No	No	No	Yes	Yes
Diabetes mellitus	No	No	No	No	Yes	Yes
Acidosis	No	No	No	No	Yes	No
Obesity	No	No	No	No	Yes	Yes
FiO_2_ > 0.35 (or > 4 L/min)	No	No	No	No	Yes	No
Tachypnoea	No	No	No	No	Yes	No
Sepsis	No	No	No	No	Yes	No
Aspiration	No	No	No	No	Yes	No
Shock	No	No	No	No	Yes	No
High risk trauma	No	No	No	No	Yes	No
BMI	No	No	No	No	Yes	No
Amiodarone	No	No	No	No	Yes	Yes
Statins	No	No	No	No	No	Yes
ACE-I/ARB	No	No	No	No	No	Yes
Sex	No	No	No	No	No	Yes
Restrictive lung disease	No	No	No	No	No	Yes
GERD	No	No	No	No	No	Yes
Cirrhosis	No	No	No	No	No	Yes
Procedure dependent						
Elective or emergency procedure	Yes	Yes	Yes	Yes	No	No
Duration of surgery	Yes	Yes	Yes	Yes	No	No
Type of surgical procedure	Yes	Yes	Yes	No	Yes	Yes
Type of anaesthesia	No	No	No	Yes	No	No
Use of supraglottic device	No	No	No	Yes	No	No
Desaturation	No	No	No	Yes	No	No
Need of vasoactive drugs	No	No	No	Yes	No	No
Mechanical ventilation characteristics	No	No	No	Yes	No	No
Validation						
Prospective external validation	Yes	No	No	Yes	No	Yes

*ASA* American Society of Anesthesiologists, *BMI* Body mass index, *ACE-I* Angiotensin converting enzyme inhibitors, *ARB* Angiotensin receptor blockers, *GERD* Gastro-esophageal reflux disease

A simpler stratification tool feasible at the bedside before emergency surgery is the early calculation of the SpO_2_/ FiO_2_ ratio within 6 h from hospital admission, which has shown to be independently correlated with ARDS development in patients at risk [20]. Several preventive strategies have been proposed to reduce the perioperative risk in patients undergoing non-urgent surgical procedures, such as smoking cessation and physical therapy [21]. However, these strategies cannot be applied to patients that require emergency procedures; therefore, risk stratification is essential in these patients. We believe and suggest that scores should be adopted and included in local hospital guidelines in the evaluation of the patient before surgical intervention to optimize the clinical and organizational pathways in the postoperative period.

#### Surgical procedure and timing

Emergency surgical procedures are by definition characterized by an elevated number of unpredictable factors that might precipitate patient’s conditions. Therefore, modifiable risk factors should be identified and managed appropriately, including timing and choice of interventions [22].

Several surgical procedures and techniques are at higher risk, such as open versus laparoscopic, upper abdominal incision, longer procedures and those requiring general anaesthesia and neuromuscular blockade [7, 17, 23]. In a large cohort of surgical patients, emergency procedures performed during night-time were independently associated with higher incidence of intraoperative adverse events and PPCs [24]: this might suggest that delaying interventions when feasible might improve surgical outcomes.

While the choice of surgical procedure and timing is typically perceived as an exclusive prerogative of the surgeon, we recommend that these factors should be discussed in team, possibly opting for less invasive and shorter duration procedures and procrastinating non-emergency procedures [25].

### Intraoperative management

#### Anaesthesia strategies in patients at risk of developing ARDS

No clear evidence is available concerning the ability of specific anaesthesia techniques to prevent the development of postoperative ARDS.

In 2016, a meta-analysis reported a non-significant trend towards less PPCs and complications in patients undergoing volatile general anaesthesia for non-cardiac surgery, as compared with intravenous anaesthesia [26]. From a theoretical point of view, volatile agents can reduce pulmonary vasoconstriction, and carry a protective effect on ischaemia-reperfusion injury [27]. On the other hand, they can potentially reduce arterial oxygenation by causing myocardial depression and low cardiac output [28]. Therefore, we cannot conclude that volatile anaesthesia is associated with less PPC than intravenous anaesthesia.

The use of locoregional anaesthesia techniques is often advocated, based on the rationale that sparing sedative drugs avoids impairment of the respiratory function; however, this concept has been challenged in specific surgical populations [29]. Moreover, these techniques can rarely be used in emergency setting. Nonetheless, a potential beneficial effect on the incidence of postoperative pneumonia and respiratory failure has been shown when general anaesthesia plus neuraxial blockade techniques are used in conjunction, especially in thoracic, abdominal and lower-limb procedures and in patients with pre-existing pulmonary disease [30]. There is a rationale suggesting the use of a peripheral nerve block when possible, thus avoiding the use of general anaesthesia and opioid consumption. Therefore, PPC may reduce, however further studies are required to corroborate these data [31]. Phrenic nerve palsy and pneumothorax are a rare but important side effects of upper-limb locoregional approaches, thus limiting their use in in frail patients [32]. In short, we cannot conclude that regional anaesthesia may result in less complications than general anaesthesia [29]. In patients with established ARDS admitted to the ICU who require emergency surgery, the same sedative drugs used for sedation in the ICU can be maintained as well as mechanical ventilation strategies, increasing the dose of narcotics and analgesics during the surgical procedure [33].

We recommend careful choice of anaesthesia technique in all patients undergoing emergency surgery, avoiding general anaesthesia if possible.

#### Intubation for general anaesthesia

Patients with established ARDS often come to the operating room already intubated and mechanically ventilated. However, the anaesthesiologist might face the challenge of managing the airway of critically ill patients also in the operating room. The team must be prepared for gas exchange impairment during induction: the intubation procedure must be performed swiftly, with readily available second choice and emergency devices. Video-laryngoscope might offer some advantage as first-choice device, but requires specific training [34]. Haemodynamic impairment should also be expected in critically ill patients, therefore, invasive monitoring, fluids and vasoactive drugs should be readily available [35].

Preoxygenation with non-invasive positive pressure improves end-expiratory lung volume and gas-exchange, allowing more time for a safe airway management procedure [36]. Before intubation, a brief period of pre-oxygenation, using pressure support of 10 cmH_2_O and PEEP of 5 cmH_2_O, as initial parameters, have been suggested. Moreover, in the emergency setting, FiO_2_ levels dispensed to the patients during pre-oxygenation should be up to 100%, especially in those with impaired respiratory function [37]: in this setting, the potential harms of hyperoxia are largely overwhelmed by the advantages of increasing the time-to-desaturation [38]. In all patients, orotracheal intubation should be performed using an endotracheal tube of the largest possible diameter according to the gender and size of the patient, to decrease the resistance of the airway and favour secretions management [39]. Respiratory outcome may also be negatively influenced by inadequate airway secretion clearance and aspiration of pharyngeal or gastric secretions, especially in the emergency setting [40].

#### Intraoperative mechanical ventilation in patients with or at risk of ARDS

There is lack of evidence regarding the best ventilator settings in patients with or at risk of ARDS in the specific setting of emergency surgery. However, optimization of mechanical ventilation with the use of protective ventilation is important to minimize VILI and improve outcome in patients with ARDS [41] and those at risk of ARDS undergoing surgical procedures [42].

While tidal volume (V_T_) size reduction is a widely accepted strategy to reduce VILI in ARDS [43] and surgical [44] patients, other ventilation parameters are under debate. There are controversies concerning the use of high PEEP levels [45] to open the lungs based on findings of physiological [46] and clinical studies [47]. In fact, authors question the beneficial effects of lung recruitment in both ARDS patients [48] and those at risk of ARDS [49]. They suggested keeping lung resting if atelectasis is tolerated and oxygenation is kept under acceptable values.

#### Ventilation modes and spontaneous breathing

Volume- (VCV) or pressure-controlled (PCV) mode can be applied unrestrictedly in ARDS patients with no influence on outcome [50], and no clear advantage in surgical patients [42]. However, observational data concerning patients at risk of developing PPCs showed that, during surgery, VCV might offer more benefits than PCV [51]. Alternative modes of mechanical ventilation include airway pressure release ventilation and high frequency ventilation, but the lack of outcome data preclude a recommendation on their routine use [35]. While spontaneous breathing activity should be suppressed in severe ARDS, it might have a protective role in mild ARDS [52] and high risk of ARDS surgical patients.

#### Tidal volume and plateau pressure during surgery

Over the last years, the concept of protective mechanical ventilation-including the use of low V_T_ to minimize barotrauma and lung injury and maintain low plateau pressure (Pplat), lower driving pressure (ΔP) with moderate levels of PEEP and the use of recruitment manoeuvres (RM) has gained particular attention, showing a positive effect on the reduction of PPCs as well as improvement outcome in ARDS patients [53] and those at risk of ARDS undergoing surgical procedures [44].

In patients with ARDS, it is recommended to maintain V_T_ of 6 ml/kg predicted body weight (PBW) [53]. However, V_T_ as low as 4–5 ml/kg, could be preferable if an adequate gas exchange is ensured, and does not increase the risk of atelectasis [54]. In fact, a small RCT showed that atelectasis do not increase with low tidal volumes and without PEEP during surgery [55]. On the other hand, a recent clinical trial comparing 4–6 ml/kg to 8–10 ml/kg PBW, and keeping a plateau pressure below 21 cmH_2_O found no benefit from lower V_T_, in terms of ventilator-free days, hospital stay and mortality in critically ill patients without ARDS [56]. Nonetheless, tidal volume is considered the main determinant of ventilator-induced lung injury and should be targeted to maintain plateau pressure < 30 cmH_2_O and low ΔP < 15 cmH_2_O. In patients with increased intra-abdominal pressure, higher values might be tolerated, correcting the upper thresholds as P_plat target, corrected_ = Pplat_target_ + (IAP – 13)/2 [57].

A recent experimental study in rats, investigating the impact of different V_T_ levels and respiratory rates on lung function, found that V_T_ was able to predict important increase in the alveolar inflammatory markers, and even maintaining low mechanical power, high V_T_ resulted in VILI [58].

In surgical patients at risk of ARDS, higher thresholds of V_T_ and lower ΔP are often considered acceptable compared to ARDS patients [42], but we recommend using the lowest pressures and volumes able to keep gas-exchange in a safe range.

#### Inspired fraction of oxygen

Inspired fraction of oxygen should aim to maintain SpO_2_ between 88 and 95% in ARDS [59] and above 92% in at risk of ARDS surgical patients [42]: higher thresholds might result in hyperoxia especially in at risk of ARDS patients, with potential detrimental effects on alveolar damage, endothelial inflammation and mitochondrial dysfunction through increasing oxidative stress and direct lung injury [60, 61]. If during surgery hypoxemia develops, FiO_2_ should be increased, followed by increase of PEEP and then stepwise recruitment manoeuvres. However, high oxygen levels during surgery can cause high risk of major respiratory complications.

Although further research on the effects of hyperoxia is needed, we suggest to target FiO_2_ levels to normoxaemia in all surgical patients undergoing emergency procedures [60].

#### PEEP titration in patients with or at risk of ARDS undergoing emergency surgery

Level of PEEP is another relevant component of lung protective ventilation. In patients with ARDS undergoing surgery, PEEP choice should be guided by the ARDS network low PEEP table [43], while latest clinical trials performed in at risk of ARDS surgical patients demonstrated that the application of low tidal volume (6–8 ml/kg) and low PEEP (< 2 cmH_2_O) reduce the risk of developing PPCs and of haemodynamic impairment [62]. No strategy for PEEP titration was demonstrated to be superior to the low PEEP ARDS network table, and experts recently made a recommendation for high PEEP (≥15 cmH_2_O) only in patients with moderate to severe ARDS, as rescue strategy [48, 53]. In this context, recruiting the alveoli de-recruits the capillaries. Thus, at higher PEEP hemodynamic is impaired and vasoactive drugs and/or more fluids are needed, which can promote further lung injury in patients at risk and worsen lung function in ARDS patients.

Patients with established ARDS previously admitted to the ICU should continue the protective ventilation received in the intensive care setting, a strategy now made feasible by modern anaesthesia machines [63]. Therefore, we suggest that PEEP should be considered as a tool to maintain oxygenation between 88 and 95%. We recommend using the minimal PEEP level ensuring adequate gas-exchange in all patients undergoing emergency surgery, considering higher PEEP levels only as rescue therapy in severe ARDS.

#### Recruitment manoeuvres

The effects of recruitment manoeuvres on clinical outcomes in patients with ARDS remain uncertain. Recruitment manoeuvres have received a conditional recommendation in patients with ARDS [53]; among them, stepwise manoeuvres are recommended [64]. However, the Alveolar Recruitment for ARDS trial (ART) comparing lung recruitment and titrated positive end-expiratory pressure compared with low PEEP in patients with ARDS showed an increased 28-day all-cause mortality in the maximal recruitment strategy [47]. In surgical patients without ARDS, recruitment manoeuvres reduced the incidence of PPCs only when combined with V_T_ reduction [62, 65], and in a recent report in obese patients [66] their use performed by squeezing the anaesthesia bag was associated with an increased incidence of PPCs. However, during laparoscopic surgery alveolar recruitment manoeuvres followed by positive end-expiratory pressure improved lung function and reduced postoperative pain [67].

We do not recommend a routine use of recruitment manoeuvres in patients with and without ARDS undergoing emergency surgical procedures, considering them only as a rescue strategy in the presence of refractory gas-exchange impairment.

#### Haemodynamic and fluid management

In patients at risk of developing ARDS, maintenance of an adequate tissue perfusion often requires a huge amount of fluids. However, fluid overload and positive balance in patients with ARDS increase extravascular lung water level and mortality [68]. Moreover, blood products transfusions can further increase the risk of ARDS and worsen endothelial lung damage. A restrictive fluid strategy should be therefore applied with haemoglobin trigger for transfusion of > 7 g/dl [69]. Albumin can be used to reduce fluid leakage from the capillary into the alveolus mediated by increased intravascular oncotic pressure. Moreover, in a state of shock, vasopressor could be considered to optimize mean arterial pressure avoiding fluid overload [70], in order to maintain a mean arterial pressure value greater than 65–70 mmHg, that is essential in patients with shock to provide organ perfusion. However, in ARDS patients this might be challenging because of the haemodynamic instability which often occurs during anaesthesia in this subgroup of patients. In addition, haemodynamic instability can be related to increased hypoxic pulmonary vascular resistance or due to the pathology itself. Systemic inflammation can have a direct negative effect on cardiac contractility and function, thus resulting in left ventricle impairment. Right heart failure is common, and its function could be further impaired by the application of mechanical ventilation with high PEEP and intrathoracic pressure with reduced preload. Moreover, right heart dilation could impair left ventricular filling and preload. Left ventricular dysfunction further increase pulmonary capillary hydrostatic pressure and subsequent extravascular lung water extravasation [68].

We recommend using a conservative fluid strategy and conservative transfusion threshold in both ARDS patients and those at risk of ARDS during emergency surgery. Patients with ARDS frequently have hemodynamic impairment requiring specific monitoring to target fluid, vasoconstrictors and inotropes administration. While pulmonary artery catheter (PAC) is the gold standard, its use in the ICU has dramatically decreased over the last decades; the use in the OR is limited to very specific settings, such as cardiac surgery or surgery in patients already admitted to the ICU with a PAC previous placed. Although PAC has been almost abandoned both in OR and ICU, it remains an excellent instrument for the diagnosis and management of several critically illness like pulmonary hypertension, cardiogenic shock and unexplained dyspnea [71].

Modern minimally invasive monitoring systems have replaced its use, but have several limitations [72]. Basic monitoring requires an arterial line, while more critical patients might benefit from non-calibrated or calibrated pulse contour monitors, to estimate cardiac output and vascular resistances. Chest ultrasound can investigate both the lungs and the cardiac function, and is increasingly being used in the emergency setting [73], helping to discriminate between respiratory and cardiac causes of gas exchange impairment, and to detect pulmonary hypertension or right ventricular failure, often reported in ARDS patients [74].

#### Pharmacologic strategies

Regarding the pharmacological intraoperative management, neuromuscular blocking agents should be used cautiously in patients who can be extubated after surgery, because if not appropriately reversed could result in postoperative residual curarisation and increased incidence of PPCs [2]. Furthermore, Kirmeier E. et al. found that the association between the use of neuromuscular blocking agents and PPCs is probably dose-independent, and even a single dose such as that used for intubation could promote respiratory function impairment [23]. Avoidance or limited use of opioids is feasible in most surgical procedures, and might offer benefits in particular in patients at high risk but planned for extubation after surgery [75], in obese patients and those with suspected or confirmed obstructive sleep apnoea syndrome [76].

### Postoperative management

Planned ICU admission is suggested after emergency surgery that is associated to higher risk of complications, but criteria are poorly standardised and planned ICU admission was not associated with better outcome in elective surgery [77]. While ICU admission is obvious for ARDS patients, criteria for planned or unplanned admission in subjects at risk of ARDS undergoing emergency surgery are less clear. Specific indications to ICU admission could be based on clinical reasoning, mechanical ventilation requirement, need for respiratory and cardiac monitoring, difficult glycaemic control, intraoperative surgical or anaesthetic complications and organ failure [78].

To decrease the risk of respiratory complications, there are several postoperative strategies that could be adopted: head-up or sitting position, encouragement of deep breathing exercises, early mobilization, intensive physiotherapy, incentive spirometry [79], airway toilette careful fluid management and an adequate opioid-sparing analgesia. However, high-quality evidence for these strategies is lacking in both elective and emergency surgery. Non-invasive positive pressure ventilation can be used to treat early mild ARDS, but its role as prophylactic measure in patients with previously healthy lungs at risk of ARDS is unclear [80].

Pulmonary infections and pneumonia are the most common cause of pulmonary ARDS [81]. Early recognition of underlying respiratory infections and pneumonia should include the identification of the causative pathogens, with eventually early empiric antibiotic therapy and subsequent de-escalation to directed therapy in patients with sepsis [82]. They may need intensive treatments that require the critical care setting.

When ARDS is established, it should be managed according to international guidelines, and while treating the underlying conditions, when identifiable. Continuous monitoring of vital parameters after surgery allows prompt identification of complications at their earlier stage, in particular pulse oximetry which is still underused [23].

## Conclusions

ARDS is a life-threatening condition, which can occur in the perioperative period in the critically ill surgical patients. Early recognition and treatment are necessary in this context to reduce mortality and morbidity. Specific intraoperative anaesthesiologic management and in particular the use of lung protective ventilation are first line strategies to meet the goals of alveolar protection and avoid further lung damage. Preventive strategies including a careful risk stratification of the patients and preoperative optimization of the clinical conditions, can significantly reduce the occurrence of pulmonary complications and prevent the development of ARDS. Patients at high risk or already affected by ARDS should be managed in the intensive care unit in the postoperative phase.

## Data Availability

Not applicable

## Abbreviation

ARDS: Acute respiratory distress syndrome
DP: Driving pressure
ICU: Intensive care unit
LIP: Lung injury prediction score
PBW: Predicted body weight
PCV: Pressure-controlled ventilation
PEEP: Positive end-expiratory pressure
PPC: Postoperative pulmonary complication
Pplat: Plateau pressure
RM: Recruitment manoeuvre
SLIP-2: Surgical lung injury prediction 2 model
VCV: Volume-controlled ventilation
VILI: Ventilator-induced lung injury
V_T_: Tidal volume
